# Effects of gut microbiota on immune responses and immunotherapy in colorectal cancer

**DOI:** 10.3389/fimmu.2022.1030745

**Published:** 2022-11-08

**Authors:** Xinxin Hou, Zongmei Zheng, Jiao Wei, Ling Zhao

**Affiliations:** Academy of Integrative Medicine, Shanghai University of Traditional Chinese Medicine, Shanghai, China

**Keywords:** colorectal cancer, gut microbiota, inflammation, immune response, immunotherapy

## Abstract

Accumulating evidence suggests that gut microbial dysbiosis is implicated in colorectal cancer (CRC) initiation and progression through interaction with host immune system. Given the intimate relationship between the gut microbiota and the antitumor immune responses, the microbiota has proven to be effective targets in modulating immunotherapy responses of preclinical CRC models. However, the proposed putative mechanisms of how these bacteria affect immune responses and immunotherapy efficacy remains obscure. In this review, we summarize recent findings of clinical gut microbial dysbiosis in CRC patients, the reciprocal interactions between gut microbiota and the innate and/or the adaptive immune system, as well as the effect of gut microbiota on immunotherapy response in CRC. Increased understanding of the gut microbiota-immune system interactions will benefit the rational application of microbiota to the clinical promising biomarker or therapeutic strategy as a cancer immunotherapy adjuvant.

## Introduction

Colorectal cancer (CRC) is the third most common cancer and the second leading cause of cancer-related death with more than 1.9 million new cases and 935,000 deaths estimated to occur in 2020 worldwide (1). Although risk factors are recognized as western dietary patterns, excess body weight, and lifestyle factors including heavy alcohol consumption and cigarette smoking, the specific underlying pathogenetic mechanisms are still not fully-elucidated.

The gut microbiota has been discussed widely over the past decades which plays an important role in all the different phases of CRC process from oncogenesis to metastasis, from treatment to prognosis prediction (2, 3). Investigators have identified specific microbial features relevant to CRC, data from cross-sectional epidemiological studies and unbiased microbiome profiling of stools and colorectal tissues have uncovered specific bacterial taxa that contribute to CRC (4). Mechanistic insights into a microbe’s contributions to carcinogenesis support that gut microbiota alterations induce genotoxin production, inflammation, metabolic regulation, and local and systemic immune response, thereby influencing the development of CRC (5). Immune escape or suppression has long been proposed to constitute a critical step in both tumor formation and progression (6). Studies have highlighted that intact mucosal immunity maintains a balanced bacterial composition in the gut, whereas disruption of this immunological circuit, either prior to, or as a consequence of tumor development, accelerates CRC initiation and progression (7, 8). In addition, some specific bacterial taxa are shown to regulate immunotherapy responses in both animal models and human cohorts (9–12). In this sense, researchers are focusing on the deep and complex relationship between microbiota and immune regulation to better understand cancer biology and to formulate novel therapeutic approach. In this review, we outline the clinical findings of changes in gut microbiota composition in CRC patients, highlight potential mechanisms of gut microbiota-modulated immune responses and the possible impacts on responses to immunotherapy in CRC.

## Clinical findings of microbial changes in CRC patients

Next-generation sequencing studies have revealed the potential association of microbial compositional changes (dysbiosis) within CRC patients, including increased proportions of *Fusobacterium nucleatum*, *Bacteroides fragilis*, *Escherichia coli*, and *Streptococcus*, *Peptostreptococcus* species (13–15). These findings raised the possibilities for clinical applications using gut microbiota analysis as screening, prognostic or predictive biomarkers. Moreover, deciphering key microbiome signatures within different stages of cancer progression may offer possibilities for treatment stratification and metastasis surveillance (16). The gut microbiota changes across all stages of CRC patients were summarized in **Table 1**. As indicated, dynamic shifts in microbial composition in gut microbiota were observed during multistep CRC progression.

**Table 1:** Summary of microbial changes across stages of CRC.

References	Specimens	Sample size	Stage I	Stage II	Stage III	Stage IV
(15)	Stool	Healthy (n = 251) MP (n = 67) S0 (n = 73) SI/II (n = 111) SIII/IV (n = 74)	↑ *Gemella morbillorum*		↑ *Fusobacterium nucleatum*, *Colinsella aerofaciens*, *Dorea longicatena*, *Porphyromonas uenonis, Selenomonas sputigena*, *Streptococcus anginosus*, *Desulfovibrio vietnamensis*, *Bilophila wadsworthia*	
(16)	Tissue	Healthy (n=61) Adenoma (n=47) Carcinoma (n=52)	↑ *Fusobacterium*, *Parvimonas*, *Gemella*, *Leptotrichia* ↓ *Bacteroides*, *Blautia, Faecalibacterium prausnitzii*, *Sutterella, Collinsella aerofaciens*, *Alistipes putredinis*		—	—
(17)	Stool	Healthy (n=358) Adenoma (n=42) S0-II (n=47) SIII-IV (n=44)	↑ *Fusobacterium nucleatum, Peptostreptococcus stomatis, Porphyromonas asaccharolytica;* ↓ *Eubacterium Rectale, Eubacterium eligens*, *Streptococcus salivarius*		—	—
(18)	Stool	Healthy (n=45) CRC (n=53)	↑ *Enterobacteriaceae*, *Fusobacterium nucleatum;* ↓ *Lactobacillus, Bifidobacterium, Clostridium cluster* I		—	—
(19)	Tissue	SI (n=7) SII (n=37) SII (n=31)	↑ *Peptostreptococcus*, *Parvimonas*	↑ *Fusobacterium, Streptococcus*, *Parvimonas*, *Burkholderiales*, *Caulobacteraceae*, *Delftia*, *Oxalobacteraceae*	↑ *Fusobacterium*, *Burkholderiales*, *Caulobacteraceae*, *Oxalobacteraceae*, *Faecalibacterium*, *Sutterella*	—
(20)	Tissue Stool	S0 (n=8) SI-II (n=97) SIII-IV (n=73)	—	—	↑ *Peptoclostridiu*, *Akkermansia;* ↓ *Gelria*	
(21)	Tissue	Dysplasia (n=3) Adenocarcinoma (n=15)	—	—	↑ *Fusobacteria, β-Proteobacteria*	
(22)	Stool	Healthy (n=178) SIII-IV (n=74)	—	—	↑ *Fusobacterium nucleatum*, *Peptostreptococcus stomatis*, *Gemella morbillorum*, *Parvimonas micra*	
(23)	Stool	Healthy (n=187) SI-II (n=74) SIII-IV (n=63)	—	↑ *Fusobacterium nucleatum, Parvimonas micra*		
(24)	Tissue	SI-II (n=12) SIII-IV (n=11)	↑ *Escherichia, Halomonas, Shewanella*, *Granulicatella, Lactobacillus*		↑ *Bacteroides, Prevotella, Peptostreptococcus, Streptococcus, Ruminococcus, Fusobacterium, Akkermansia;* ↓ *Granulicatella, Lactobacillus*	
(25)	Tissue	SI-II (n=32) SIII-IV (n=48)	↑ *Dietzia*, *Paludibacter, Porphyromonadaceae*, *Propionibacterium*		↑ *Granulicatella, Coprococcus*, *Phycisphaeraceae*	
(26)	Stool	Healthy (n=30) SI-II (n=19) SIII-IV (n=23)	↑ *Peptostreptococcus, Collinsella Ruminococcus, Parvimonas*, *Peptostreptococcus*	↑ *Hydrogenoanaerobacterium*	↑ *Akkermansia*	↑ *Phascolarctobacterium, Parasutterella*, *Comamonas, Cloacibacillus, Olsenella;* ↓ *Escherichia-Shigella, Alistipes, Blautia, Eisenbergiella, Intestinimonas, Eggerthella, Anaeroglobus*
(27)	Stool	Healthy (n=91) SI-II (n=39) SIII-IV (n=32)	↑ *Klebsiella quasipneumoniae*, *Klebsiella oxytoca*, *Klebsiella variicola*		↑ *Faecalibacterium prausnitzii*, *Bacillus cereus*, *Lactococcus species*	

MP, multiple polypoid adenomas with lowgrade dysplasia; S0, intramucosal carcinoma, stage 0/pTis CRC; SI, stage I CRC; SII: stage II CRC; SIII, stage III CRC; SIV, stage IV CRC.↑, increase in the abundance; ↓, decrease in the abundance.

## Gut microbiota elicits tumor-promoting inflammation

It is well-known that chronic inflammation is a risk for CRC initiation and development. Overall, 2.2 million new cancer cases were attributable to infections by different etiological agents, including viruses, bacteria and parasites in 2018, representing 13% of all cancer cases (28). The bacterium enterotoxigenic *Bacteroides fragilis* (ETBF) is a significant source of chronic inflammation and has been implicated as a risk factor for CRC, which can up-regulate spermine oxidase (SMO)-dependent generation of reactive oxygen species (ROS) and induce inflammation, leading to DNA damage in colonic epithelial cells (29). Diverse cytokines like tumor necrosis factor (TNF)α, interlukin (IL)-6, IL-1β, IL-23, and IL-17, can be triggered by microbes or their products, contribute to the progression of intestinal tumorigenesis (30). Upon invading the stroma, bacteria can trigger both innate responses *via* recognition through pattern recognition receptors (PRRs), eliciting secretion of a repertoire of cytokines and chemokines. *Streptococcus gallolyticus* has long been associated with colonic pathologies. Although a causal relationship to CRC is not clear, increased production of inflammatory factors, including cyclooxygenase (COX)-2, IL-1 and IL-8, in *Streptococcus gallolyticus*-bearing tumor tissue might indicate its possible contribution to tumor progression (31). *Fusobacterium nucleatum* has been reported to drive a pro-inflammatory intestinal microenvironment through metabolite receptor -dependent modulation of IL-17 expression in *Apc^min/+^* mice (32). *Fusobacterium nucleatum* colonization leads to increased intestinal short chain fatty acid (SCFA) levels and human CRC tissues harboring *Fusobacterium nucleatum* are primed to sense these immunomodulatory metabolites, with higher expression of the genes encoding the SCFA receptors *FFAR2* and *NIACR1*. Furthermore, in *Ffar2* ^-/-^ mice, *Fusobacterium nucleatum* failed to increase Th17 cell frequency, suggesting that *Fusobacterium nucleatum* shaped Th17 response is FFAR2-dependent (32). The inflammasome NACHT, LRR, and PYD domains-containing protein 3 (NLRP3) is a global sensor of pathogen-associated molecular pattern molecules (PAMPs) and damage-associated molecular patterns (DAMPs) and its activation leads to secretion of both IL-1β and IL-18. Studies have provided evidence to indicate that the gut microbiota serve as critical modulators of inflammasome activity and susceptibility to the development of intestinal inflammation and cancer (33). Indeed, the presence of some of the cytokines (IL-17, IL-6, IL-1β, TNFα) also correlate with poor prognosis in CRC patients (34). The chronic activation of inflammatory signals not only suppresses adaptive immune responses but simultaneously supports tumor growth, *via* mechanisms such as the increased release of growth and immunomodulatory factors (35). For example, tumor infiltrating myeloid cells release growth factors, such as epidermal growth factor (EGF), hepatocyte growth factor (HGF), and fibroblast growth factor (FGF) (36, 37). These growth factors can support cancer cell proliferation, survival, motility, and invasion, by triggering the activation of MAPK, Wnt/β-catenin or PI3K/AKT/mTOR signaling pathway, thus facilitate CRC progression (38). The continuation of inflammatory responses and tumor progression to malignancy on the one hand, changes the composition of immune cells from immune activators to immune suppressors, on the other hand, potential cytokines and chemokines facilitate the recruitment of immune cells with immunosuppressive functions, such as myeloid-derived suppressor cells (MDSCs) and regulatory T cells (Tregs) (39), which eventually maintains tumor cell survival and immune escape (discussed below). In addition, chronic inflammation is now accepted as a major influence in the outcome of CRC, treatment with anti-inflammatory (such as aspirin) mitigates CRC progression and extends patient survival (40).

## Gut dysbiosis facilitates immune escape in CRC

Here, we outline the potential mechanisms of microbial-modulated immune escape from innate and adaptive immune perspectives and discuss dysbiosis-immune interaction during CRC progression.

### Modulating innate immune surveillance against tumors

The immune system has an extraordinary capacity to recognize and respond to a range of microbial patterns and danger signals. The first cells to fight microbes are the myeloid derived innate immune cells (41). Bacterial species that translocate through the epithelial barrier induce recruitment of myeloid cells. Through their PPRs, these cells recognize microbes, thus influencing the type and intensity of innate and adaptive immunity (42). Dysbiosis can enhance gut responsiveness to bacteria and its products, however during chronic infections, it may lead to a miscoordination between inflammation and immune suppression, thus favors tumor growth. In fact, an important feature of tumors is the generation, development, and expansion of myeloid cells with special immunosuppressive properties including tumor-associated neutrophils (TANs), tumor-associated macrophages (TAMs), regulatory dendritic cells (DCs), and MDSCs (43). For example, *Fusobacterium* promotes the growth of colorectal cancer through the induction of the activity of MDSCs and TAMs (44, 45). Tumor invading pathogen bacteria also trigger the activation of neutrophils that infiltrate into tumor stroma, whereby the earliest tumor-infiltrating neutrophils may serve to inhibit expansion of colon microbiota to limit tumorigenesis and progression (46–48). However, established tumors may evolve a more pro-tumorigenic TAN phenotype and elicit the production of tumor-promoting cytokines (49). Neutrophils can also produce ‘neutrophil extracellular traps’ (NETs), upon activation to ensnare and neutralize pathogens. Recent studies highlight the function of NETs in cancer progression and metastasis, NETs are able to wake up dormant cancer cells promoting cancer relapse, and are able to entrap circulating cancer, thus enhancing metastasis spread (50). Bacteria derivations also play a role in escalating the tumor-associated immune suppression. Tryptophan-derived microbial metabolites activate the aryl hydrocarbon receptor in TAMs to suppress anti-tumor immunity (51). Gut microbiota tend to produce butyrate, which in turn can inhibit the DCs’ antigen presentation (52, 53). Beyaz et al. showed that high fat diets (HFDs) resulted in changes in the composition of the gut microbiota (54), and in a Kras-driven mouse model of intestinal cancer, HFD-altered gut microbiome, which, in turn, resulted in reduced major histocompatibility complex (MHC) II expression on DCs and engages in immune evasion (55), suggesting that dietary in association with the gut microbiota, are critical modulators to the development of intestinal cancer. In addition to the aforementioned myeloid cells, another example is the inhibitory effect of *Fusobacterium nucleatum* on natural killer (NK) cells. *Fusobacterium nucleatum* produces the Fap2 protein, which binding to the inhibitory receptor T cell immunoglobulin and ITIM domain (TIGIT) on NK cells, thus directly inhibiting cell-killing of tumor cells (56). Yet, we have very little insight into the roles of microbiota on innate immune cell populations, further studies are needed to interrogate the precise functional contributions of gut microbe on these innate immune cell subsets.

### Reprogramming adaptive anti-tumoral responses

With improved tools, recent work has suggested two broad categories of bacteria-related tumor escape of adaptive immune attack: 1. Microbes influence anti-tumor effectors directly by serving as antigens which mediate recognition by host T cells (57, 58), 2. Microbes facilitate the resistance of immune attack through the immune suppressive pathways such as inducing immune exhaustion (59, 60).

Intestinal microbiota has been proposed to induce commensal-specific memory T cells that cross-react with tumor-associated antigens. Indeed, memory responses by CD4+ and CD8+ T cells specific for *Enterococcus hirae*, *Bacteroides fragilis*, and *Akkermansia muciniphila* are associated with favorable clinical outcome in cancer patients (11, 57, 58), suggesting that microbe-specific T lymphocytes may contribute to anti-tumoral immune responses. The optimal recognition of the antigen induces a specific activation of T cells, thereby driving T cell activation and differentiation of CD4+T cell subsets into Th1, Th2, and Th17 or Tregs (61, 62). It should be noted that dysbiosis induced T cells are capable of switching their phenotypes, which in turn set the proclivity to inflammatory, immunostimulatory or immunosuppressive reactions depending on tumor context and specific bacteria. The modulation by distinctive microbiome antigens can also consequently affect the activation of cytotoxic CD8+ T cells that limit the direct lysis of cancer cells (63).

In cancer, like in chronic infection, the long exposure to the antigen leads to a dysfunction of T cells, represents the state of “exhaustion” (64, 65). In the early stage of azoxymethane (AOM)/dextran sulfate sodium (DSS) mice model, gut dysbiosis (increased *Prevotellaceae* and decreased *Anaeroplasmataceae*) promoted tumorigenesis by stimulating CD8+ T cells activation, durable hyperstimulation of CD8+ T cells resulted in T cell exhaustion, leading to increased tumor susceptibility (66). Microbiota can also provoke sustained expression of the inhibitory molecules, such as cytotoxic T lymphocyte antigen 4 (CTLA-4), T cell immunoglobulin and mucin domain-containing protein 3 (TIM3), programmed cell−death protein 1 (PD−1), or the ligand PD-L1, which are the most prominent examples of immune−checkpoint molecules underlying immune−escape mechanisms (60). The FAS/FAS ligand (FASL) apoptotic pathway is also highly relevant to immune evasion, which induces apoptosis of lymphocytes (67). Histopathological analyses have revealed that FASL is upregulated in metastatic tumor compared the primary tumor in patients with CRC (68). Thus, simultaneous loss or downregulation of FAS and upregulation of FASL on tumor cells might contribute to tumor evasion of immune−mediated cytolysis. The microbiota-derived SCFAs, such as butyrate, can be absorbed across the intestinal epithelium and exert their influence on T cells *via* G-protein-coupled receptors (GPRs). Butyrate was shown to promote cellular metabolism, enhance memory potential of activated CD8+ T cells through promoting mitochondrial function and cellular metabolism (69). Taken together, these findings reveal a role for the microbiota in the modulation of T cell responses in CRC which may have important implications on immunotherapy.

As another important adaptive immune cell type, B cells perform immune surveillance as antigen presenting cells (APCs) or function by stimulating immunoglobulins (Ig A) and producing cytokines (IL-10, TGFβ, often terms as regulatory B cells, Bregs) (70). Changes in gut microbiota composition and a diverse role of B cells have been implicated at the mucosal interface. *Helicobacter hepaticus* colonization has been shown to relieve tumor burden in CRC mice and increase B cells maturation and infiltration (71). *Parvimonas micra* was also shown to be closely associated with the antigen-presenting HLA-DR (+) B cells in a CRC cohort (72). In another study of familial adenomatous polyposis (FAP), loss of resident memory T cells and γδ T cells, excess IgA antibody secretion and increased IgA+ peripheral B cells were found to accompany intestinal microbial dysbiosis, implicating mucosal immune dysfunction as a contributing factor in the etiology of CRC (73). Above studies highlighted the interactions between microbiota and B cells in CRC, but the underlying mechanism remains largely unresolved. Some recent reports have shed light on the microbial metabolites, SCFAs and microbial tryptophan catabolites to regulate B cell activation and antibody responses (74–76). More recently, Wang et al. found that leucine-tRNA-synthetase-2 (LARS2)-expressing B cell (LARS B) with TGF-β1-dominant feature correlates with shortened survival in CRC, mechanistically, LARS2 programmed mitochondrial nicotinamide adenine dinucleotide (NAD+) regeneration and oxidative metabolism, thus determining the regulatory feature of LARS B cells in which the NAD-dependent protein deacetylase sirtuin-1 (SIRT1) was involved (77).

**Figure 1:**
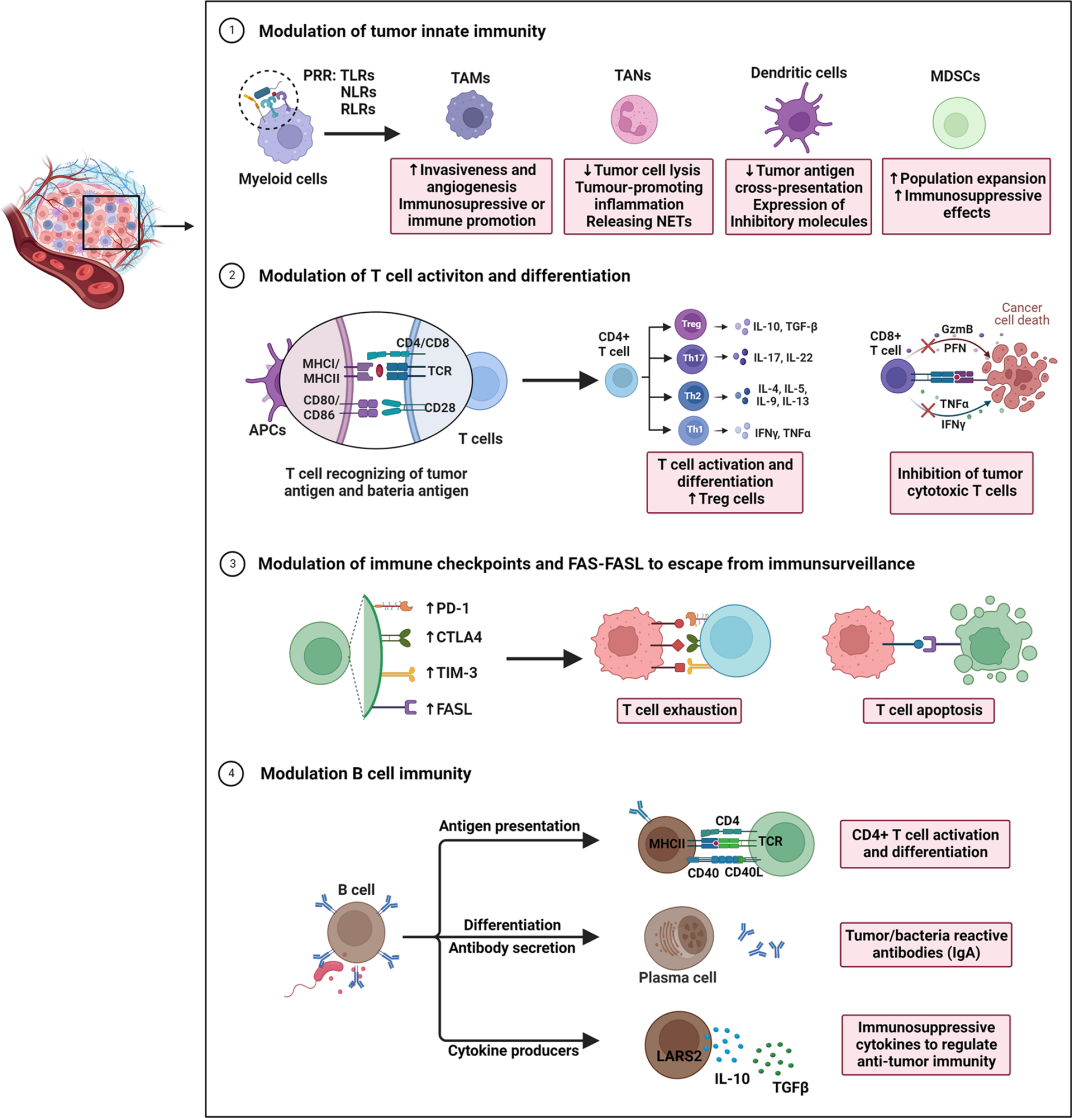
Overview of gut microbiota-modulated immune responses in tumor progression. The underlying actions and mechanisms by which the microbiota affects tumor immune escape are summarized as follows: 1) Under pathogenic conditions (dysbiosis), bacterial species that translocate through the epithelial barrier induce recruitment of myeloid cells. Myeloid cells recognize microbes *via* their pattern recognition receptors (PPR). During this process, the immunosuppressive mechanisms observed in the tumor microenvironment (TME) are activated underlie chronic infections. 2) Intestinal microbiota and intra-tumoral bacteria can be directly presented by antigen-presenting cells, thereby driving T cell activation and differentiation of CD4+T cell subsets into Th1, Th2, and Th17 or Tregs. Microbiota may also inhibit the dendritic cells’ antigen presentation, consequently decreasing the CD8+T cell response. 3) By modulating T cell immune checkpoint receptor-ligand pairs (e.g., CTLA-4/CD80/CD86 and PD1/PD-L1/PD-L2), they impact T cell exhaustion, and directly impacting efficacy of immune checkpoint inhibitors. Additional mechanisms of immune escape include expression of the apoptotic proteins FAS/FASL to induce immune cell apoptosis. 4) Gut microbiota may also regulate B cell infiltration, development, and polarization. These B cells exert anti/pro-tumor immunity through acting as APCs to reshape T cell responses, secreting tumor/bacteria-specific antibodies (IgA), and producing cytokines (IL-10, TGFβ), all of which are associated with immune processes in CRC.

Overall, the immune impact of gut microbiota on CRC partially depends on shaping innate and adaptive immune responses (e.g., suppressing immunosurveillance, inducing T cell exhaustion and apoptosis, etc.), consequently leading to immune escape (**Figure 1**). In future, clarification of possible role of microbiota in modulation of populations and functions of innate and adaptive immune cells, as well as the crosstalk between different types of immune cells remain important research areas.

### Role of gut microbiota in shaping an immune-privileged (pre-) metastatic niche

**Figure 2:**
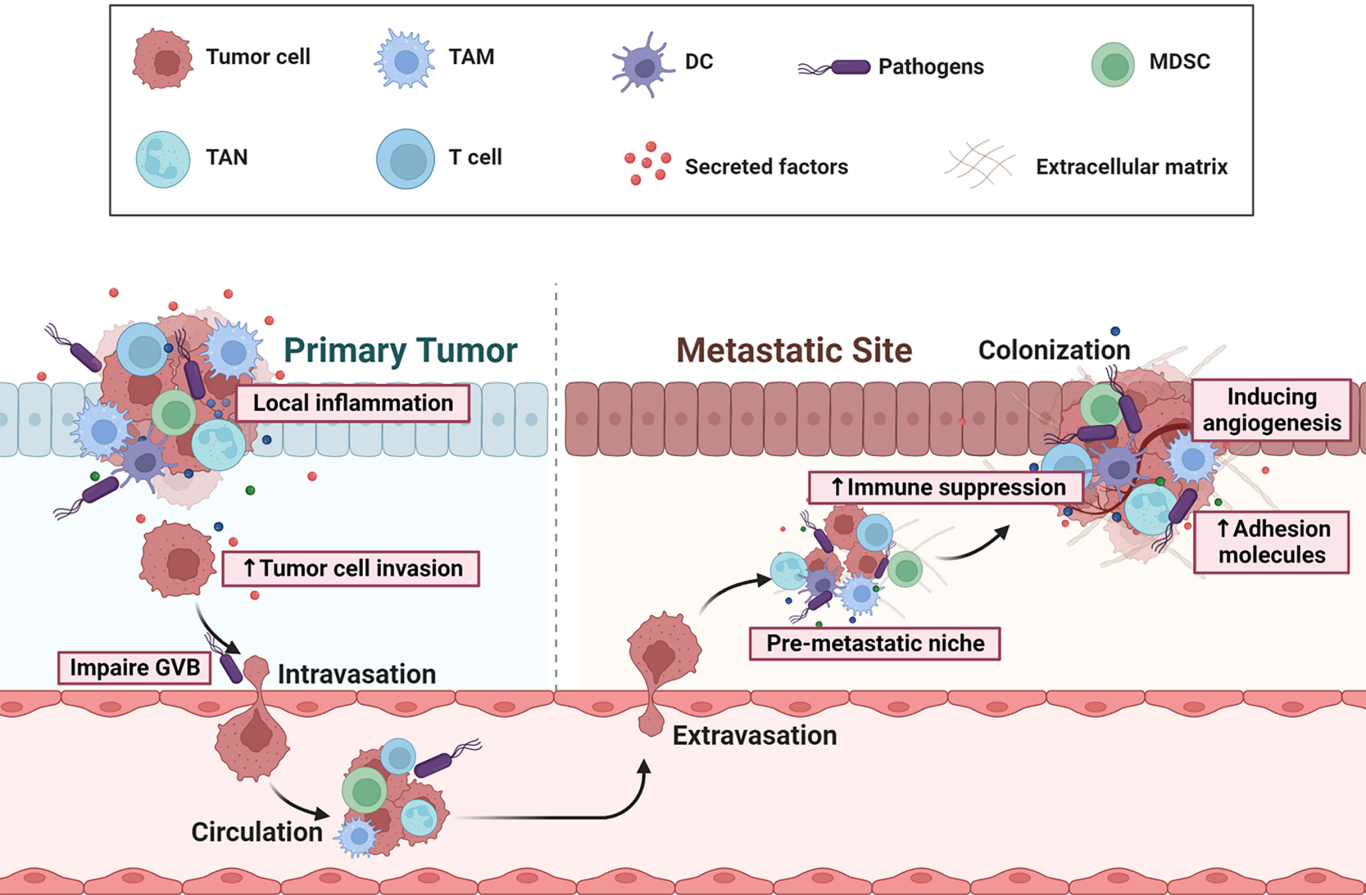
Overview of gut microbial dysbiosis on CRC initiation and metastasis. Dysbiosis can cause a chronic, pro-inflammatory milieu, by eliciting secretion of a repertoire of cytokines or growth factors, and thereby facilitates tumor cell invasion, adhesion, extravasation and survival. Some pathogens (eg: *Escherichia coli*) can induce gut vascular barrier (GVB) disruption, which allows bacteria to reach the distant organ and initiate the recruitment of immune cells with immunosuppressive functions, such as MDSCs and Tregs. Thus, the microbiota can contribute to the immune escape of distant tumors, the pre-metastatic niche maturation, adhesion, angiogenesis, and eventually the formation of metastasis.

Although studies have observed the presence of microbiota in metastatic liver or lung organs of CRC patients, the underline mechanisms by which microbiota affects CRC metastases formation are only now being uncovered. In the secondary sites, immunosuppressive cell types, such as TAMs and MDSCs populate (pre) metastatic niches, where they help direct metastatic dissemination by creating a niche that is permissive to tumor colonization (78). These cells have been shown to achieve these pro-tumoral functions by (1) generating a proinflammatory milieu (2), remodeling the matrix and creating a pro-angiogenic, pro-invasive environment (3), maintaining an immunosuppressive microenvironment, and (4) secreting growth factors that maintains the growth of metastatic cells. Pathogen *Escherichia coli* can upregulate Cathepsin K (CTSK) expression which serves as a vital mediator between the imbalance of intestinal microbiota and CRC metastasis (79). CRC-secreted CTSK stimulates CRC progression through accelerating M2 polarization of TAMs in a TLR4-mTOR-dependent pathway. Meanwhile, cytokines (IL-10, IL-17) secreted by activated M2 macrophage, in turn, promote CRC cells invasion and metastasis by activating NF-κB pathway (79). *Fusobacterium nucleatum* can boost liver metastasis by modulating liver microenvironment featured with accumulation of MDSCs, and reduction of NK and Th17 cells (80, 81). *Peptostreptococcus anaerobius* was also reported to induce chronic inflammation and modulate tumor microenvironment by recruiting MDSCs, TANs and TAMs (82). More recently, Bertocchi et al. demonstrate that *Escherichia coli* induces gut vascular barrier (GVB) disruption, which allows bacteria to reach the liver and initiate the recruitment of inflammatory cells, contributing to pre-metastatic niche maturation and favoring metastases formation (83). These results demonstrate that host microbiota acts as a key modulator during CRC metastasis by facilitating (pre-) metastatic niche formation which support cancer cells seeding in secondary organs (**Figure 2**). Re-education of the metastatic niche, through alterations in metastasis-related bacteria and associated pathways, may have favorable consequences for metastatic CRC therapy.

## Microbiota influence response of cancer immunotherapy

Cancer immunotherapies, specifically immune checkpoint inhibitors (ICIs) PD-1/PD-L1 and CTLA-4, have become effective strategies for cancer treatment (84). The reciprocal interactions between gut microbiota and cancer immune response raised the possibility that gut microbiota could significantly influence cancer immunotherapy response. In CRC, microbiota-based methods to enhance immunotherapy efficacy has not yet been demonstrated in human cohort. However, in animal models, specific bacterial species have been shown associated with immunotherapy response, including *Bifidobacterium* spp (9, 85). *Bacteroides fragilis* (10), *Akkermansia muciniphila* (11, 86), and *Alistipes shahii* (12). In Microsatellite Stability (MSS)-type CRC tumor-bearing mice, changes in gut microbiome affected the expression of immune-related cytokines IFN-γ and IL-2 in the tumor microenvironment, resulting in a different therapeutic effect of PD-1 antibody, and *Prevotella_sp._CAG:485* and *Akkermansia* may maintain the normal efficacy of PD-1 antibody (87). Mager et al. investigated the efficacy of ICIs therapy in both AOM/DSS induced colitis-associated cancer and MC38 tumor-bearing models, they found that *Bifidobacterium pseudolongum*, *Lactobacillus johnsonii*, and *Olsenella species*—that significantly enhanced efficacy of anti-PD-L1 and anti-CTLA-4 (88). Another study identified that tumors in antibiotic-treated or germ-free mice did not respond to CTLA-4 blockade, oral gavage of *Bacteroides thetalotaomicron*, *Bacteroides fragilis*, *Burkholderia cepacia*, or the combination of *Bacteroides fragilis* and *Burkholderia cepacian* recovered the anticancer response to CTLA-4 Ab following antibiotic treatment in mice subcutaneously engrafted with MC38 cells (10). Tanoue et al. isolated a consortium of 11 bacterial strains that is capable of enhancing therapeutic efficacy of ICIs in subcutaneous MC38 tumor models (89). Furthermore, gut microbiota was shown to impact immunotherapy efficacy related to innate responses. Researchers have observed that systemic administration of *Bifidobacterium* converts the nonresponder mice into responders to anti-CD47 immunotherapy and improves the antigen-presenting capacity of DCs (90). Song et al. found that an engineered LPS-targeting fusion protein significantly boosts anti-PD-L1 therapy against CRC tumors (91), suggesting that anti-LPS treatment may promote anti-PD-L1 immunotherapy for mouse model of CRC. Collectively, these results indicate that the gut microbiota could be used to develop new therapeutic strategies to enhance CRC immunotherapy response. Recently, a human clinical trial (NCT04729322) which studies the effect of fecal microbiota transplant and re-introduction of anti-PD-1 therapy (pembrolizumab or nivolumab) for the treatment of metastatic CRC in anti-PD-1 non-responders is under recruiting. Although still under investigation, we are beginning to exploit the tremendous potential of the gut microbiota to predict immunotherapy response, and to enhance immune surveillance for a more precise immunotherapeutic intervention.

## Conclusions and perspectives

It is important to decipher the specialized roles of gut microbiota in regulating the immune response in cancer, as the current landscape of the gut microbiota-host immune axis has expanded from basic research to clinical development (92). We have gained insights into the gut microbiota dysbiosis in CRC patients (**Table 1**). However, considerable challenges remain, for example, although multiple studies have identified specific bacteria that are associated with CRC, inconsistency across these studies exists. This may due to diverse life styles, various diet patterns and different disease stages, since gut microbiome varied substantially according to these factors (93). More studies mining of clinical large cohort data, omics, and preclinical models are needed to facilitate consensus for potential characteristics of bacterial alternation and to determine whether such changes are a cause or an effect in CRC development. Additionally, although we have deepened our view on the innate and adaptive immune responses modulated by gut microbiota in CRC (**Figure 1**), owing to the complex relationship between commensal and pathogenic microbes and host immunity, more detailed studies of the sophisticated network between gut microbiota and host immune system are required. In CRC, the individual heterogeneity between patients in the response to ICIs is largely associated with the gut microbiota composition, suggesting that manipulation of gut microbiota could improve immunotherapy responses (2). Therapeutics that target microbiota is explored in conjunction with cancer immunotherapies such as FMT, prebiotics, probiotics, Chinese traditional medicine, and dietary approaches (94–97). At present, microbial intervention is mainly performed in preclinical studies at the animal level, whereas it is not yet tested with large samples in the context of clinical trials and more clear mechanisms of effective microbiota to enhance immune surveillance and influence immunotherapy responses remain unknown in CRC.

In summary, substantial efforts must be devoted to pursue a deeper understanding of the mechanistic links and to exploit for clinical benefit. The insight gained into the specialized functions of the microbiota on immunity and cancer will help to apply gut microbiota-based strategies into the clinical anti-tumor adjuvant therapies, particularly in the context of conjunction with existing immunotherapies.

## Author contributions

XH and LZ conceived the study. XH, ZZ, and JW screened the literatures and wrote the manuscript. LZ reviewed the manuscript. All authors contributed to the article and approved the submitted version.

## Funding

The work is supported by the Shanghai Rising-Star Program (22QA1408700 to LZ), National Natural Science Foundation of China (82104955 to XH) and China Postdoctoral Science Foundation (2022M712154 to XH).

## Conflict of interest

The authors declare that the research was conducted in the absence of any commercial or financial relationships that could be construed as a potential conflict of interest.

## Publisher’s note

All claims expressed in this article are solely those of the authors and do not necessarily represent those of their affiliated organizations, or those of the publisher, the editors and the reviewers. Any product that may be evaluated in this article, or claim that may be made by its manufacturer, is not guaranteed or endorsed by the publisher.
